# Combining PARP Inhibition, Radiation, and Immunotherapy: A Possible Strategy to Improve the Treatment of Cancer?

**DOI:** 10.3390/ijms19123793

**Published:** 2018-11-28

**Authors:** Mathieu Césaire, Juliette Thariat, Serge M. Candéias, Dinu Stefan, Yannick Saintigny, François Chevalier

**Affiliations:** 1 LARIA, iRCM, François Jacob Institute, DRF-CEA, 14076 Caen, France; mathieu.cesaire@hotmail.fr (M.C.); saintigny@ganil.fr (Y.S.); 2 UMR6252 CIMAP, CEA-CNRS-ENSICAEN-Université de Caen Normandie, 14076 Caen, France; 3 Radiotherapy Unit, Centre François Baclesse, 14000 Caen, France; j.thariat@baclesse.unicancer.fr (J.T.); d.stefan@baclesse.unicancer.fr (D.S.); 4 ProMD, Chemistry and Biology of Metals Laboratory, Univ. Grenoble Alpes, CEA, CNRS, BIG-LCBM, 38054 Grenoble, France; serge.candeias@cea.fr

**Keywords:** immunotherapy, PARP inhibitors, radiotherapy, antitumor immune response, combined therapies

## Abstract

Immunotherapy has revolutionized the practice of oncology, improving survival in certain groups of patients with cancer. Immunotherapy can synergize with radiation therapy, increase locoregional control, and have abscopal effects. Combining it with other treatments, such as targeted therapies, is a promising means of improving the efficacy of immunotherapy. Because the value of immunotherapy is amplified with the expression of tumor antigens, coupling poly(ADP-ribose) polymerase (PARP) inhibitors and immunotherapy might be a promising treatment for cancer. Further, PARP inhibitors (PARPis) are being combined with radiation therapy to inhibit DNA repair functions, thus enhancing the effects of radiation; this association might interact with the antitumor immune response. Cytotoxic T lymphocytes are central to the antitumor immune response. PARP inhibitors and ionizing radiation can enhance the infiltration of cytotoxic T lymphocytes into the tumor bed, but they can also enhance PD-1/PDL-1 expression. Thus, the addition of immune checkpoint inhibitors with PARP inhibitors and/or ionizing radiation could counterbalance such immunosuppressive effects. With the present review article, we proposed to evaluate some of these associated therapies, and we explored the biological mechanisms and medical benefits of the potential combination of radiation therapy, immunotherapy, and PARP inhibitors.

## 1. Introduction

### 1.1. The Immune System in Tumor Control

Tumors interact with the immune system in a dynamic process, leading to a balance between the shaping and control of the tumor by the immune system [1]. Tumor-infiltrating lymphocytes (TILs) have important functions in tumor control and are being investigated with regard to predicting the tumor response to immunotherapies [2]. In particular, CD8+ T lymphocytes are central to the antitumor immune response [1], and their tumor-infiltrating capacity correlates with patient survival [3]. The infiltration of specific activated CD8+ lymphocytes against tumor antigens (CTLs) induces a fundamental response against the tumor; thus, TILs are key to controlling tumor proliferation [4]. Tumor-associated antigens (TAAs) that are recognized by T lymphocytes induce this specific immune response [5].

The immune system has important functions in the development and control of a tumor. Cancer immunosurveillance comprises 3 phases: elimination, equilibrium, and escape [3]. In this process, IFNγ and lymphocytes prevent the development of a primary tumor [6]. During the elimination phase, the immune response induces an effective extrinsic tumor-suppressor system. However, this event also leads to the immuno-selection of tumor cells that are better able to survive in an immunocompetent host, namely, the escape phase. Between these phases lies an equilibrium phase, in which tumor growth remains under pressure by the immune system. The innate and adaptive immune systems are involved in controlling the tumor. At the interface between innate and adaptive immunity, the cytokine IFNγ has pleiotropic functions—notably in the activation of natural killer (NK) cells and CTLs [3]. IFNγ signaling upregulates MHC I and MHC II expression and increases the presentation of TAAs to lymphocytes. IFNγ also induces specific TAA-activated CD8+ lymphocytes and, thus, an antitumor response that is mediated by CTLs [7]. The infiltration of CTLs correlates with a good prognosis in many tumors [8,9].

Ionizing radiation may increase the generation of TAAs and their presentation through the increased expression of major histocompatibility complex (MHC) molecules [10]. Ionizing radiation improves the immune response and synergizes with immunotherapies [11]. TAAs probably result from tumor mutations. Tumors with a high mutational burden might respond better to immunotherapies [12]. Cytotoxic molecules that target DNA repair functions, such as poly(ADP-ribose) polymerase inhibitors (PARPis), could enhance the mutational load in tumors with a pre-existing deficiency in DNA repair function [13]—a concept that has been demonstrated in mismatch repair (MMR)-deficient colorectal cancers [14,15]. Thus, PARPis and ionizing radiation might improve the efficacy of such immunotherapies as those using immune checkpoint inhibitors.

### 1.2. Combination of Treatments with Immunotherapies

Immune checkpoint inhibitors are important tools in this approach. Immune checkpoint inhibitors target negative regulatory immune surface molecules, such as cytotoxic T lymphocyte-associated antigen 4 (CTLA-4) and the programmed cell death protein 1 pathway (PD-1/PDL-1) [16]. CTLA-4 was the first immune checkpoint receptor to be targeted clinically.

T cell activation requires the interaction between a T cell receptor (TCR) and antigen-bound MHC; it also needs co-stimulatory signals, such as those provided by the interaction between CD28 on the T cells and B7 on the antigen-presenting cell (APC). Early after activation, CTLA-4 translocates to the cell membrane to downregulate T cell activation and maintain immunological homeostasis. CTLA-4 interacts with B7 and initiates regulatory signals, leading to T cell inhibition [17,18]. In melanoma, the CTLA-4 antibody ipilimumab was the first therapy to improve patient survival [19]. In clinical practice and in many clinical trials, anti-CTLA-4 immunotherapies, such as ipilimumab and tremelimumab, are used in many tumor models [16].

Another immune checkpoint is the PD-1/PDL-1 axis. The T cell receptor PD-1 downregulates T cell activation to control immunological homeostasis. PDL-1 is expressed on many cell types, such as various immune cells, mesenchymal support cells, and vascular cells. The upregulation of PDL-1 by tumor cells confers on them resistance to the immune system and allows them to escape immunosurveillance mechanisms [20]. To mitigate this immune evasion, anti-PD-1/PDL-1 treatments have been clinically developed for many tumors, such as non-small-cell lung cancer (NSCLC) [21], melanoma, and renal cell cancer. These therapies have resulted in an objective response in 20% to 25% of patients [22]. Thus, immunotherapies can clearly add to our arsenal of antitumor treatments. However, even though they proved to be very efficient when they work, the main challenges are currently to improve this efficiency (i.e., treatments result in tumor regression in a larger proportion of treated patients) and to increase their field of application (i.e., to show efficiency against more tumor types). These goals might be achieved by—or at least might benefit from—the association of immunotherapies with other treatments, such as the use of PARPis and/or radiation exposure to increase tumor immunogenicity and make it a better target for the immune system, especially CTLs, once the immune checkpoints are relieved.

**Figure 1:**
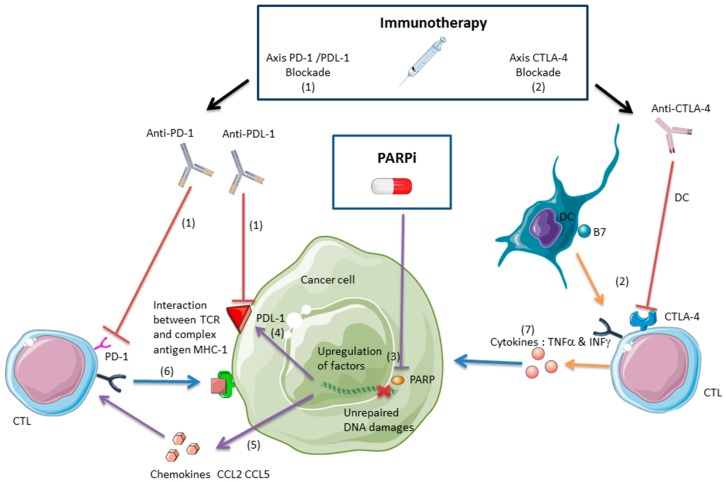
Poly(ADP-ribose) polymerase inhibitors (PARPis) and immune checkpoint inhibitors synergize and enhance an antitumor immune response mediated by specific activated CD8+ T lymphocytes against tumor antigens (CTLs). PARPis promote tumor-infiltrating lymphocytes (TILs) through the upregulation of chemokines and induce an immune response mediated by CTLs. However, PARPis induce the upregulation of PDL-1, inhibiting CTLs and promoting immune tumor escape. The anti-CTLA-4 immunotherapy activates T cells and can synergize with PARPis to induce an antitumor immune response. The anti-PDL-1/PD-1 immunotherapy can reverse the CTL inhibition mediated by the PDL-1 upregulation induced by PARPis. Thus, anti-PDL-1/PD-1 can synergize with PARPis to induce an antitumor immune response. [Arrows represent the activation or the induced expression processes of PARPi (violet), immune cells (orange), cytotoxicity (blue). T bars represent the inhibition processes of PARPi (violet) and immune check point inhibitors (red).]

**Figure 2:**
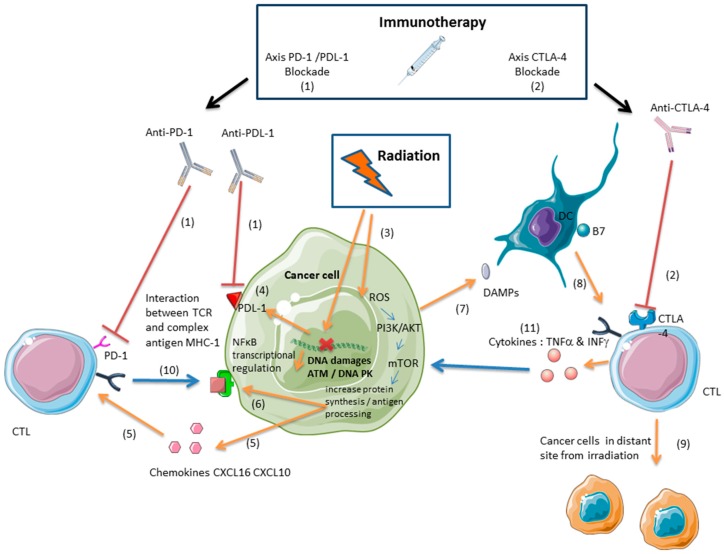
Ionizing radiations and immune checkpoint inhibitors synergize to enhance an antitumor immune response mediated by specific activated CD8+ T lymphocytes against tumor antigens (CTLs). [Arrows represent the activation or the mechanisms induced by radiation (orange), and cytotoxicity (blue). T bars (red) represent the inhibition processes of immune check point inhibitors.]

To improve the efficacy of immune checkpoint inhibitors, many compounds such as anti-angiogenic agents, due to the relationship between angiogenesis and immunosuppression [23], and other targeted therapies are being tested in combination with immunotherapies [24]. Recently, PARPis were suggested to function in antitumor immunity. There is potential synergy between PARPis and immune checkpoint inhibitors (Part II, Figure 1). Current clinical trials are evaluating the safety and efficacy of treatments that combine PARPis and immune checkpoint inhibitors in various types of cancer (Tables S1 and S2). Another strategy for enhancing the antitumor immune response that is mediated by CTLs concerns the addition of radiation therapy (Part III, Figure 2). We report the clinical trials that have evaluated the combination of radiation and immunotherapy in several types of cancer (Table S3). The following combination of PARPis and radiation therapy is presented as part 4 of this review (Table S4). In this review, we discuss the various approaches that can be used to improve or recover a CTL-based immune response after the combination of PARPis and radiation therapies with the use of immune checkpoint inhibitors.

## 2. Interactions and Synergy between PARPis and Immunotherapies in Tumor Control

### 2.1. PARPis as a Cytotoxic Treatment

Poly(ADP-ribose) polymerase (PARP) proteins catalyze the polymerization of poly(ADP-ribose) on proteins. This reversible post-translational modification of proteins, also called parylation, has been implicated in many cellular mechanisms, notably DNA repair. There are 17 distinct proteins that have been identified as members of the PARP family in mammals, but only PARP-1, PARP-2, and PARP-3 are known to be involved in DNA repair pathways, such as homologous recombination (HR) and conventional (c-NHEJ) and alternative non-homologous end joining repair (alt-NHEJ) [25].

PARP detects single-strand breaks (SSBs) and, through its parylation activity, recruits proteins that mediate DNA repair, such as XRCC1, which stabilizes the DNA break; DNA polymerase β, which performs complementary base synthesis; and DNA ligase III, which ligates the ends of DNA [26]. Ultimately, the auto-parylation of PARP releases it from the SSB site. PARP activity is enhanced in many tumors [27]. Thus, the inhibition of PARP activity is being used increasingly as a therapeutic strategy. In certain instances of PARP inhibition, PARP remains on the SSB site and blocks the recruitment of DNA repair proteins, leaving the SSB unrepaired.

In the treatment of cancer, the cytotoxic effects of PARPis have various mechanisms, such as competition with NAD+ for the C-terminal catalytic site of the PARP and the trapping of PARP-DNA complexes, which prevents the recruitment of DNA repair proteins. Synthetic lethality results in the accumulation of unrepaired DNA damage and ultimately leads to cell death [28]. PARPi-induced synthetic lethality is particularly efficient in cancers that are deficient in the HR DNA repair pathway, such as those with BReast CAncer (BRCA) mutations. Synthetic lethality is based on the synergy between two mechanisms that are not lethal individually.

The inhibition of PARP effects the accumulation of unrepaired DNA damages, primarily SSBs. During replication, SSBs induce the formation and accumulation of double-strand breaks (DSBs) in DNA, but deficiencies in HR prevent their repair. In such cases, the NHEJ repair pathway can be used to repair DSBs, but this process introduces more errors than HR. Thus, PARPi-induced synthetic lethality in tumors that express PARP causes the accumulation of DSBs, which are toxic during DNA replication [28]. PARPis are currently approved for BRCA-deficient ovarian cancer [29], and phase III trials are ongoing to determine their efficacy in triple-negative breast cancer (TNBC) [30]. PARPis rarely have cytotoxic effects alone, but they can initiate an artificial synthetic lethality mechanism when combined with other drugs, such as platins [31].

### 2.2. PARPis and the Antitumor Immune Response

The effects of PARPis on the immune system are not well defined. PARP-1 upregulates proinflammatory cytokines, such as TNFα and IL-6, through the activation of NF-κB; thus, PARPis downregulate these cytokines, reducing local inflammation [32,33]. PARP-1 and its inhibition maintain the balance between proinflammatory and anti-inflammatory responses [34]. PARP-1 regulates dendritic cell (DC) maturation from monocytes; PARP inhibitors reduce the expression of CD86 and CD83 and reduce the expression of IL-12 and IL-10 [35]. Exogenous addition of IL-12 could restore the expression of CD86. Thus, PARPis can play a role in DC maturation and function depending on the cytokine expression [34,35]. PARP-1 modulates the activation of nuclear factor of activated T cell (NFAT) which is essential in T cell function. Thus, PARPis cause an increase in NFAT-dependent transactivation and a delay in NFAT nuclear export [36]. PARPis can protect CD8+ T lymphocytes against phagocytes-derived oxygen radicals [37]. PARPis protect CD8+ lymphocytes from oxygen radical-induced apoptosis [38]. Thus, PARPis might be suitable for tumors with significant infiltration of CTLs that are treated by ionizing radiation in association with immunotherapy.

In a mouse model that has been inoculated with the BR5FVB1-Akt cell line, a BRCA1-deficient ovarian cancer, PARPis induce a local immune response [39]. The number of peritoneal cytotoxic CD8+ T cells and NK cells rises, and they produce more IFNγ and TNFα. In addition, the percentage of myeloid-derived suppressor cells (MDSCs) decreases. MDSCs promote tumor progression, notably through T cell suppression [40]. Thus, PARPis promote an antitumor immune response by increasing TILs, such as NK cells and CTLs, and lowering MDSC levels. In this model, the upregulation of CCL2 and CCL5 could explain the increase in TILs [39], as CCL2 can induce TILs in ovarian cancer [41]. The capacity of PARPis to influence the composition and function of TILs should be examined in association with immunotherapies to enhance the response. The advantages of coupling PARPis and immunotherapies have been reported in several mice models [39,42,43].

The development of immunotherapies and PARPis for certain types of cancer has led to the study of their combination. Most of the cancers that have been tested with these combinations are tumors that lack DNA repair function, including BRCA-deficient tumor cells (Table S1). These tumors respond well to PARPis [25] and immune checkpoint inhibitors, such as anti-PD-1 [14]. Hypermutated tumors are potential targets for immune checkpoint inhibitors, increasing their expression of TAAs to promote specific immune responses [44]. In melanoma patients who are treated with anti-PD-1, high mutational loads are associated with improved survival, and tumors from responding patients are enriched for mutations in DNA repair functions, such as BRCA-2 [45]. These data support the rationale for combining immune checkpoint inhibitors and PARPis for tumors with impairments in DNA repair.

These data are summarized in Figure 1. The interest in such therapeutic combinations has led to clinical studies on their safety and efficacy. We have listed the clinical trials that have been registered at clinicaltrials.gov to display the different types of targeted tumors (Table S1). In these combinations with immunotherapies, PARPis are listed in Table S2.

(1) Anti-PD-1/PDL-1 immunotherapies avoid the interaction between the PDL-1 on the tumor cell surface and the PD-1 on the T cell surface, allowing the CTL activation against the tumor [20].

(2) Anti-CTLA-4 immunotherapies enhance the activation of CD8+ T lymphocytes against the tumor. CTLA-4 blockade inhibits the interaction between CTLA-4 on the T cell surface and the B7 on the dendritic cell (DC) membrane, inducing an immune response mediated by CTLs [17]. Anti-CTLA-4 synergizes with PARPis in a BRCA-deficient ovarian cancer model and the combination treatment improves mice survival [42].

(3) PARPis inhibit PARP activity and augment unrepaired DNA damages such as single-strand break (SSB) and double-strand break (DSB) and induce tumor cell death [28].

(4) PARPis can induce the upregulation of PDL-1, but this immunosuppressive effect can be counterbalanced by anti-PDL-1/PD-1 immunotherapies. Blockade PDL-1 synergizes with PARPis in a breast cancer model, and the combination inhibits tumor growth proliferation and improves mice survival [43].

(5) PARPis induce the upregulation of chemokines such as CCL2 and CCL5 in BRCA-deficient ovarian cancer mice models. Chemokines recruit CTLs and the CTL infiltration induces an antitumor immune response, inhibiting the growth tumor proliferation and improving mice survival [39].

(6) Intra-tumoral CTLs exert a direct cytotoxic effect through the interaction between their T cell receptor (TCR) and the complex MHC1-antigen [1].

(7) Intra-tumoral CTLs exert an indirect cytotoxic effect through TNFα and INFγ [46,47].

#### 2.2.1. Anti-CTLA-4 and PARPis

In vitro, IFNγ and TNFα inhibit the proliferation of BRCA-deficient cancer cell line (BR5-Akt BRCA1-) in association with PARPis (veliparib). In mouse models that are inoculated with this cancer cell line, anti-CTLA-4 and PARPis synergize to elicit a greater response than either agent alone, improving survival [42]. This response was mediated by intraperitoneal infiltration of IFNγ-producing CD8+ T lymphocytes. The increase in local IFNγ in response to the combination of anti-CTLA-4 and PARPis was sufficient to inhibit tumor growth, but this combination had no such effect on BRCA1-sufficient ovarian cancer cells that were inoculated into the mice. Thus, this study shows the benefits of blending PARPis and anti-CTLA-4 to treat BRCA-deficient ovarian cancer. Anti-CTLA-4 immunotherapies induce an antitumor immune response. This effect has long been thought to be mediated by activated antitumor T lymphocytes once their inhibition is removed by abrogation of CTLA-4-B7 interactions [48]. However, it was recently proposed that the restoration of CTL activity and subsequent antitumor immune response in pre-clinical models rather resulted from anti-CTLA-4-mediated depletion of immunosuppressive regulatory T cells (Tregs) in the tumor bed [49]. Whether this mechanism also operates during immunotherapy in humans is currently debated. It was indeed reported that anti-CTLA-4 immunotherapy may increase the ratio of effector to regulatory T cells, that is, alleviate the immunosuppressive activity of regulatory T cells, without the depletion of Tregs [50]. Nevertheless, the antitumor activity of anti-CTLA-4 immunotherapy was attributed to IFNγ and TNFα producing CD8+ T cells in pre-clinical studies [51].

#### 2.2.2. Anti-PDL-1/PD-1 and PARPis

By influencing the function and composition of TILs, particularly CTLs, PARPis could have immunosuppressive effects or improve the antitumor response [39,42,43]. Anti-PD-1/PDL-1 could reverse the immunosuppressive effects of PARPis and synergize to elicit a greater response [43], and if PARPis enhance the immune response, anti-CTLA-4 could synergize with them and enhance this effect [42]. Anti-PDL-1, combined with PARPis, did not induce an antitumor response in Higuchi et al. [42]. However, a recent study by Jiao et al. [43] has offered new perspectives on the combination of PARPis (olaparib) and anti-PDL-1/PD-1 immunotherapies, tested in vitro and in vivo in triple-negative breast cancer (TNBC) cells. By histology, human breast cancer tissues showed an inverse correlation between the parylation of proteins and PDL-1 expression. PARPis upregulated PDL-1 on the surface of EMT6 tumor cells, a TNBC cell line, in vitro and in vivo, when inoculated into a syngeneic mouse model. This upregulation was mediated by inactivation of the GSK3β pathway and induced a decrease in TILs. Thus, PARPis have immunosuppressive effects through this decline in TILs. Anti-PDL-1 reversed the inhibition of TILs and, in combination with PARPis, enhanced the antitumor response over that of the PARPi and anti-PDL-1 alone. These data support further investigation of the combination of PARPis and anti-PDL-1/PD-1 immunotherapies [43]. A recent phase I study that coupled an anti-PD-1 immunotherapy, durvalumab, with olaparib in breast cancer patients resulted in good tolerance [52], encouraging its evaluation in patients. Veliparib has been combined with anti-CTLA-4 [42], and olaparib has been coupled with anti-PDL-1 [43]. It is notable that an immunotherapy has been incorporated in both cases. More studies are needed to determine the influence of PARPis on the antitumor immune response, based on the type of tumor and the PARPi.

## 3. Immunotherapy and Radiation: A Synergistic Effect Mediated by Cytotoxic Lymphocytes

### 3.1. Ionizing Radiation Induces an Antitumor Immune Response Mediated by Cytotoxic Lymphocytes

The possibility of combining PARPis and immunotherapies is a recent development. In contrast, the synergy between radiation and immunotherapies is well documented [53,54]. Many studies, based on animal models, have shown that radiation has an anti-tumor immune effect [11], for which CD8+ T lymphocytes are important [10]. Ionizing radiation induces proinflammatory lesions and fibrosis, mediated in part by the modulation of cytokines, in conjunction with damage-associated molecular patterns (DAMPs) [55]. Similar processes, accompanied by the infiltration of leukocytes, have been observed in cancer. Ionizing radiation activates CTLs through several mechanisms. Ionizing radiation can induce the generation of TAAs, killing tumor-specific T cells [56]. In addition, radiation exposure upregulates MHC-I molecules on tumor cells, increasing their ability to present TAAs to CD8+ lymphocytes [57]. Finally, ionizing radiation effects the release of DAMPs in the tumor bed [58]. They in turn activate macrophages and DCs, which then stimulate CTLs.

Notably, radiation elicits the secretion of high-mobility group box 1 (HMGB1) in the extracellular environment [59]. Under normal conditions, this protein is a chromatin factor in the nucleus but becomes a DAMP in the extracellular space. HMGB1 activates macrophages and DCs by binding to Toll-like receptor 4 on their surface, activating the NF-κB signaling pathway [60]. Subsequently, the DNA damage and activation of the ATM-dependent DNA damage response that are induced by ionizing radiation accelerate the activation and functional maturation of DCs through the phosphorylation of NF-κB essential modulator (NEMO) [61]. Thus, after high-dose radiation (10 Gy), activated DCs can recruit and activate tumor-specific CTLs [62].

In addition, ionizing radiation enhances leukocyte migration, including CTLs, across the endothelium by inducing chemokine secretion and upregulating adhesion molecules, such as ICAM-1 and E-selectin, on endothelial cells [63].

Chemokines are cytokines that recruit leukocytes to specific sites. They are involved in tumor progression and the antitumor response [64]. Ionizing radiation upregulates the chemokine CXCL-16, in association with an increase of TILs—particularly CTLs—in a mouse model that has been inoculated with the 4T1 breast carcinoma cell line [65]. The upregulation of CXCL-16 by the tumor and the induction of TILs improve survival in mice [66]. Radiation also induces the secretion of IFNγ and CXCL-10 in a melanoma mouse model, effecting the infiltration of CTLs, correlating with the inhibition of tumor growth and improved survival in mice [67]. Thus, the upregulation of such chemokines as CXCL-10 and CXCL-16 is important in the induction of CTL infiltration in the tumor due to radiation.

### 3.2. Radiation and Immunotherapies Can Synergize to Control the Tumor

The emergence of immune checkpoint inhibitors, such as anti-PDL-1, anti-PD-1, and anti-CTLA-4, which have good efficacy in many tumor models [20], raises the question of the pertinence and safety of their combination with radiation. Combinations of immunotherapies and radiation are well tolerated [68] but require further examination (Figure 2). Of great interest, these combinations proved to have synergistic effects against different tumor entities in different murine models. It was indeed found that radiation increased the expression of PD-L1 in the tumor microenvironment [69]. Immunotherapy therefore contributed to tumor regression by preventing the development of an immunosuppressive environment induced by radiation exposure. The up-regulation of PD-L1 expression following radiotherapy has also been observed in vivo, in patients treated for non-small-cell lung cancer [70] as well as in various tumor cell lines irradiated in vitro [71]. Thus, the use of immune checkpoint inhibitors can counteract the PD-1/PD-L1-mediated immunosuppressive effects of radiotherapy, and in many tumors, such as breast cancer [72], melanoma [73], kidney cancer [74], and colorectal cancer [74,75,76], immunotherapies and radiation synergize to inhibit tumor growth and improve survival in mice. Abscopal effects have been observed in many mouse models for several tumors, such as the RENCA (renal cell carcinoma) [74], 67NR (mammary carcinoma) [75], TSA (mammary carcinoma), B16OVA (melanoma) [73], and MCA38 (colorectal cancer) cell lines [74,75,76]. Beside this synergy with immune checkpoint inhibitors, radiotherapy can stimulate several other aspects of anti-tumor immune responses, as recently reviewed in [77], and these effects contribute to the efficiency of associated immuno- and chemotherapies. In the most favorable cases, the activation of antitumor immunity by such combinations evokes a systemic “vaccination-like” effect in distal sites. There is a promising future for a broad range of possible combinations of PD-1 checkpoint inhibitors with other cancer therapies [78]. As somatic mutation and deficiency in DNA damage repair function make good markers of predictive response to immune checkpoint inhibitors, ionizing radiations can be associated to enhance antitumor response [79]. The immune effect of ionizing radiation has led to the concept of radiation-induced tumor equilibrium (RITE), in relation with the mechanistic influencing of immune-checkpoint therapies on radiotherapy efficacy [80]. The efficiency of the anti-PD-1 antibody Durvalumab after chemotherapy and radiation therapy in stage III NSCLC within 14 days of completion of radiation therapy suggests that anti-PD-1 immunotherapy can interact in a synergistic way with ionizing radiations [81].

Coupling radiation with immunotherapies can induce a response outside of the radiation field, at a distant metastatic site. This distal response is called the abscopal effect—akin to in situ vaccination through the activation of CTLs that migrate to distal tumor sites that present a similar specific antigenic signature [56,82]. Several types of immunotherapies have been used to abolish immune checkpoints: the growth factor Flt3-Ligand, which enhances the number of available DCs [75]; anti-PD-1 [74]; and anti-CTLA-4 [76].

In these studies, immunotherapy or radiation alone did not induce a response in the secondary tumor site—their combination was necessary to produce an abscopal effect and improve survival in mice. The activation of CD8+ T lymphocytes appears to be required to promote this long-distance effect, because there was no antitumor response at distal sites in T cell-deficient mice [75].

The abscopal effect is mediated by the specific antigen signature of the tumor, and there is no response against a secondary tumor with a different histology than the primary tumor that was irradiated. In mouse models, fractionated doses (3 × 8 Gy or 5 × 6 Gy) were more effective than a single dose (20 Gy in 1 fraction) in enhancing the abscopal effect through the recruitment and activation of anti-tumor IFNγ-producing CD8+ T cells [76]. High-dose radiation (6 to 20 Gy per fraction) is more effective than low dose in inducing an immune response. Greater killing of tumor cells by T cells is observed, resulting from the autocrine production of type I IFN by DCs, which initiates the innate and adaptive immune responses [83]. Further, stereotactic body radiation therapy, using high-dose radiation with high precision, induces less extensive lymphopenia than conventional radiotherapy [84], constituting a better method of enhancing a CTL-mediated immune response. Clinical abscopal effects are rare, but there are several notable cases. The combination of CTLA-4 immunotherapy and radiation induced an abscopal effect in a patient with metastatic non-small-cell lung cancer who no longer responded to the immunotherapy alone, in whom CTL levels rose in a new non-irradiated left supraclavicular lymph node after excision [85].

A retrospective study that combined ipilimumab (3 mg/kg, every three weeks) and palliative radiation reported an abscopal effect in 11 of 23 patients with metastatic melanoma [86]. During immunotherapy, 13 and 8 patients were treated with radiotherapy for brain metastases and extracerebral metastases, respectively. The mean time to onset of the abscopal response was one month. The delay in the appearance of the response, up to several months, suggested the involvement of an immune response. There was a clear increase in progression-free survival in patients who experienced an abscopal effect: 22.4 months versus 8.3 months. A local response to radiotherapy was observed in 13 patients, including the 11 in whom the abscopal effect occurred, illustrating the importance of the local response to radiotherapy as a prognostic factor in inducing a potential abscopal effect. This study applied the combination sequentially, applying radiotherapy after the start of immunotherapy.

Many clinical trials are evaluating the combination of radiation and immunotherapy. We have listed the clinical trials that are targeting the types of tumors for which PARPis have potential benefit in conjunction with radiation or immunotherapy (Table S3). The main challenges concerning the association between radiation therapy and immunotherapy are about the efficient dose and fractionation of the irradiation to induce a synergistic immune effect [87], the toxicity [87], and the targeting of the immunosuppressive microenvironment [88].

(1) Anti-PD-1/PDL-1 immunotherapies avoid the interaction between the PDL-1 on the tumor cell surface and the PD-1 on the T cell surface, allowing the cytotoxic effect of CTLs against the tumor [20].

(2) Anti-CTLA-4 immunotherapies enhance the activation of CD8+ T lymphocytes against the tumor. CTLA-4 blockade inhibits the interaction between CTLA-4 on the T cell surface and the B7 on the dendritic cell (DC) membrane, inducing an immune response mediated by CTLs [17].

(3) Ionizing radiations induce DNA damages and production of reactive species of oxygen (ROS) and thus induce mechanisms which interact with the immune system [10].

(4) Ionizing radiations can induce the upregulation of PDL-1, but this immunosuppressive effect can be counterbalanced by anti-PDL-1/PD-1 immunotherapies [89,90].

(5) Ionizing radiations upregulate the expression of chemokines such as CXCL-10 and CXCL-16 and induce the recruitment of CD8+ T lymphocytes in the tumor bed [65,67].

(6) Ionizing radiations induce the expression of MHC1 and tumor associated antigen (TAA), which lead to the activation of CD8+ T lymphocytes against the tumor [10,57].

(7) Ionizing radiations induce the activation of DCs through the release of DAMPs [58].

(8) Dendritic cells activate CD8+ T lymphocytes against tumor after radiation [61].

(9) Ionizing radiations induce an antitumor immune response in distant sites from the radiation field through CD8+ T lymphocytes activated by DCs which lead to an abscopal effect. Ionizing radiations can induce this effect alone or additionally in association with immunotherapy [82].

(10) Intra-tumoral CTLs exert a direct cytotoxic effect through the interaction between their T cell receptor (TCR) and the complex MHC1-antigen [1].

(11) Intra-tumoral CTLs exert an indirect cytotoxic effect through TNFα and INFγ [46,47].

### 3.3. Radiation-Induced Immunosuppressive Effects Can Be Reversed with Immunotherapy

The activation of the antitumor immune response mediated by the radiation-induced immunogenic cell death can be counteracted by the immunosuppressive environment. Targeting the immunosuppressive tumor microenvironment in association with radiation therapy might be a relevant strategy [88]. Despite its function in the induction of immune responses through DC activation, the release of HMGB1 after radiation exposure can have immunosuppressive effects through the recruitment of MDSCs [91]. This immunosuppression can be reversed by combining anti-PD-1 immunotherapy and high-dose radiation, lowering MDSC levels in tumors, targeted by the cytotoxic effects of TNFα [89]. In animal models, ionizing radiation upregulates PDL-1 on tumor cells, inhibiting their interaction with T cells. Anti-PDL-1 or anti-PD-1 immunotherapy can mitigate this radiation-induced immunosuppressive effect [89,90].

Ionizing radiation enhances the infiltration of regulatory T cells (Tregs) into the tumor in mouse models [92]. Tregs dampen immune responses and thus allow tumor immune evasion. Therefore, radiation can have an immunosuppressive effect, which can be lifted with immune checkpoint inhibitors, such as anti-PDL-1. Recently, the immunoscore was validated in evaluating the prognosis in colorectal cancer, based on the composition of the immune infiltrate [8]. A radiomics approach is being developed to assess CD8+ lymphocyte infiltration [93] as a means to monitor and examine the balance between the immunosuppressive effects and antitumor immunity during treatment.

### 3.4. Inducing an Antitumor Immune Response with Proton or Carbon Ion Radiation

Ionizing radiation induces T lymphocyte death in vitro and in vivo in rats in a dose-dependent manner (from 0.5 to 8 Gy) [94]. Therefore, despite the ability of ionizing radiation to promote CTL infiltration into the tumor, the sensitivity of T lymphocytes to radiation might impede the antitumor immune response. Due to their biological effectiveness and physical properties, proton and carbon ions can be used to obtain stronger antitumor immune responses. The dose diffusion profile of the proton beam might spare bone marrow [95] and lymph nodes and reduce radiation-induced lymphopenia.

In addition to the dosimetric advantage of proton radiation, its greater biological efficacy adds value, because proton radiation and carbon ion radiation have a higher linear energy transfer than photon radiation. At the same physical dose, they have disparate biological effects, yielding a relative biological effectiveness of approximately 1.1 for proton radiation [96] and at least 2 for carbon ion radiation [97]. Thus, based on their properties, proton radiation and carbon ion radiation can induce immunological cell death [98].

Proton radiation and photon radiation upregulate surface cell molecules, such as ICAM1, HLA, the tumor-associated antigens MUC-1 and CEA, and calreticulin, to comparable extents, increasing the killing of tumor tissue by T cells [99]. Proton radiation inhibits metastasis in MDA MB 231 human breast cancer cells [100] through NF-κB signaling and in LM8 osteosarcoma cells, impeding the invasion and migration of tumor cells [101]. Carbon ion radiation can prevent metastasis without immunotherapy or any other treatment [102]. Further, abscopal effects have been reported in two patients with colorectal cancer [103]. Carbon ion radiation and proton radiation have shown potential in enhancing the antitumor immune response, based on their ability to inhibit metastasis without any additional treatment [102].

## 4. PARPis and Ionizing Radiation: A Promising Combination Therapy

Radiosensitizer molecules are used to enhance the effects of radiation on tumors, improving the antitumor response with lower toxicity. PARPis are potential radiosensitizers, based on their ability to enrich unrepaired DNA damage [28]. By suppressing HR and promoting error-prone alt-NHEJ, PARPis can intensify cell death [28]. Thus, PARPis might also suppress PARP-1-dependent c-NHEJ and radiosensitized tumor cells with deficiencies in DNA repair functions other than HR [104].

There are many encouraging studies on this combination treatment, especially for Ewing sarcoma, which presents with a EWS-FLI1 fusion transcript. EWS-FLI1 maintains the expression of PARP-1 and induces resistance to radiation. PARP-1 inhibitors and radiation synergize to increase the apoptosis of sarcoma cells in vitro and in vivo. The inhibition of PARP-1 inhibits the proliferation of cultured tumor cells and tumor growth in xenograft models [105]. In an in vitro pancreatic tumor model, PARP inhibition synergizes with radiation and augments apoptosis. In a xenograft model of pancreatic cancer, PARPis inhibited tumor growth and improved survival in mice [106].

Glioblastoma (GBM) is currently treated with radiation and temozolomide (TMZ), an alkylant chemotherapy. Despite this treatment, the patient prognosis remains poor. In a GBM mouse model, PARPis synergize with temozolomide and radiation, slowing tumor growth and promoting survival [107]. In tumor models (GBM, NSCLC, small-cell lung cancer, pancreatic cancer, colorectal cancer, locally advanced rectal cancer, head and neck squamous cell cancer, prostate carcinoma, breast cancer, cervix carcinoma), PARPis have had good efficacy as radiosensitizers, with an enhanced death ratio of between 1.04 and 2.87. Their effects included inhibition of tumor cell proliferation, decreased clonogenic survival, delayed tumor growth, and improved survival in mice [108]. We have listed the clinical trials that have examined PARPis in combination with radiation in the clinicaltrials.gov library (Table S4).

The radiosensitizing effect of this combination raises concerns about its toxicity, especially for bone marrow. The secondary hematological effects of PARPis, such as myelosuppression [109], could amplify when combined with pelvic or large-field spinal radiation. Combinations with heavy particle radiation, such as proton radiation and carbon ion radiation, could avoid this issue, because the ballistic properties of these particles allow one to spare bone marrow and reduce the myelosuppressive effects. In preliminary in vitro experiments, we observed a response by chondrosarcoma cells to the combination of radiation and PARPis [108]. Preclinical models that can be used to analyze the effects of heavy ion radiation should be developed in this setting, especially with regard to PDL-1 and chemokine expression, and the advantages of combining this treatment with immune checkpoint inhibitors should be determined. Indeed, chondrosarcomas express high levels of PDL-1; thus, immune checkpoint inhibitors have limited effects, despite the presence of a large TIL population [110,111].

## 5. Conclusions and Perspectives: A Rationale for Combining PARPis, Ionizing Radiation, and Immunotherapy

As the combination of PARPis and radiation emerges as a possible treatment, its impact on the antitumor immune response needs to be examined. PARPis and radiation can upregulate the expression and secretion of chemokines, such as CCL2, CCL5 (PARPis, [42]), CXCL-16, and CXCL-10 (radiation, [65,66,112,113]) and promote intratumoral infiltration by CTLs. The effects of their combination remain to be formally determined. PARPis [43] and ionizing radiation [89,90] can upregulate PDL-1 on the tumor cell surface, inducing an immunosuppressive effect. Thus, adding anti-PD-1/PDL-1 immunotherapies to this combination might improve the antitumor response. Therefore, the changes in immune molecule profiles that are induced by the combination of radiation and PARPis in tumoral cells and the composition of TILs, notably CTLs, should be studied to understand the underlying mechanisms. The development of animal models to study the effects of a triple therapy that combines PARPis, radiation, and immune checkpoint inhibitors or T cell therapies will be required to determine whether these approaches improve the antitumor immune response. In this context, it will be particularly interesting to compare the effects of heavy charged particles and photon radiation on the antitumor immune response and identify the advantages of using radiosensitizers with PARPis to reduce radiotoxicity.

## Supplementary Materials

Supplementary materials can be found at http://www.mdpi.com/1422-0067/19/12/3793/s1. Table S1: Clinical trials evaluating the combination of PARPi and immunotherapy in various cancer.; Table S2: Development of PARPi in association with immunotherapy; Table S3: Association between external radiation therapy and immunotherapy; Table S4: Clinical trials evaluating the combination of PARPi and radiation in various cancers.

## Abbreviations

APC	Antigen-presenting cell
BRCA	BReast CAncer
CCL2	Chemokine (C-C motif) ligand 2
CCL5	Chemokine (C-C motif) ligand 5
CTLs	specific activated CD8+ lymphocytes against tumor antigens
CTLA-4	Cytotoxic T lymphocyte-associated antigen 4
CXCL-16	Chemokine (C-X-C motif) ligand 16
CXCL-10	Chemokine (C-X-C motif) ligand 10
DC	Dendritic cell
DSB	Double-strand break
GBM	Glioblastoma
Gy	Gray
HMGB1	High-mobility group box 1
MDSC	Myeloid-derived suppressor cells
MHC	Major histocompatibility complex
NSCLC	Non-small-cell lung cancer
PARP	Poly(ADP-ribose) polymerase
PARPi	PARP inhibitor
PD-1	Programmed cell death protein 1
PDL-1	Programmed cell death ligand 1
TAA	Tumor-associated antigen
TCR	T cell receptor
TIL	Tumor-infiltrating lymphocyte
TNBC	Triple-negative breast cancer
TMZ	Temozolomide
