# Supplementary material for: Combining PARP Inhibition, Radiation, and Immunotherapy: A Possible Strategy to Improve the Treatment of Cancer?
Source: Int J Mol Sci. 2018 Nov 28;19(12):3793. doi: 10.3390/ijms19123793 (PMC6321381; doi:10.3390/ijms19123793)
Supplement: Supplementary file 1 [file ijms-19-03793-s001.zip › Supplementary Table 1.docx]

**Supplementary Table 1: Clinical trials evaluating the combination of PARPi and immunotherapy in various cancer.**

6 studies are currently evaluating the safety and the efficiency of the association between a PARP inhibitor and an immunotherapy in ovarian cancer, triple breast negative cancer, prostate cancer, and some digestive cancer such as pancreatic cancer or HER2 gastric cancer. The immunotherapies associated with PARPi are anti PD-1, anti PDL-1 or anti CTLA-4 (source: clinicaltrials.gov library).

| **ClinicalTrials.gov Identifier** | **Phase** | **Cancer** | **Drugs Immunotherapy** | **Drugs PARPi** | **Other drugs** |
| --- | --- | --- | --- | --- | --- |
| NCT02660034 | I/II | relapsed, platinum-sensitive ovarian cancer with BRCA1/2 mutations or with DNA HRD | BGB-A317, PD-1 blockade | BGB-290 |  |
| NCT02571725 | I/II | BRCA-deficient ovarian cancer | Tremelimumab, CTLA-4 blockade | Olaparib |  |
| NCT02953457 | I/II | reccurent ovarian, fallopian tube or primary peritoneal cancer | Tremelimumab, CTLA-4 blockade Durvalumab, PDL-1 blockade | Olaparib |  |
| NCT02484404 | I/II | reccurent ovarian, fallopian tube or primary peritoneal cancer | Atezolizumab, PDL-1 blockade | Olaparib | Cediranib |
| NCT03167619 | II | Advanced Triple negative breast cancer, platinum-treated | Durvalumab, PDL-1 blockade | Olaparib |  |
| NCT02849496 | I/II | Triple negative breast cancer, stage III/IV | Atezolizumab, PDL-1 blockade | Veliparib |  |
| NCT02484404 | I/II | triple negative breast cancer | Atezolizumab, PDL-1 blockade | Olaparib | Cediranib |
| NCT02660034 | I/II | triple negative breast cancer | BGB-A317, PD-1 blockade | BGB-290 |  |
| NCT02484404 | I/II | small cell lung cancer | Atezolizumab, PDL-1 blockade | Olaparib | Cediranib |
| NCT02660034 | I/II | extensive-stage disease small cell lung cancer | BGB-A317, PD-1 blockade | BGB-290 |  |
| NCT02484404 | I/II | metastatic castration-resistant cancer prostate | Atezolizumab, PDL-1 blockade | Olaparib | Cediranib |
| NCT02660034 | I/II | metastatic castration-resistant cancer prostate | BGB-A317, PD-1 blockade | BGB-290 |  |
| NCT02660034 | I/II | advanced or metastatic pancreatic adenocarcinoma | BGB-A317, PD-1 blockade | BGB-291 |  |
| NCT02484404 | I/II | colorectal cancers | Atezolizumab, PDL-1 blockade | Olaparib | Cediranib |
| NCT02660034 | I/II | HER2-negative gastric or gastroesophageal junction cancer | BGB-A317, PD-1 blockade | BGB-290 |  |
