# Supplementary material for: Combining PARP Inhibition, Radiation, and Immunotherapy: A Possible Strategy to Improve the Treatment of Cancer?
Source: Int J Mol Sci. 2018 Nov 28;19(12):3793. doi: 10.3390/ijms19123793 (PMC6321381; doi:10.3390/ijms19123793)
Supplement: Supplementary file 1 [file ijms-19-03793-s001.zip › Supplementary Table 2.docx]

**Supplementary Table 2**: Development of PARPi in association with immunotherapy.

| Name | PARP Targeted | FDA approval | Development in association with immunotherapy | Type of immunotherapy in association | FDA approval for the association |
| --- | --- | --- | --- | --- | --- |
| Olaparib  (AZD2281) | PARP 1-2 | In BRCA mutated ovarian cancer | Phase I/II and in animal model | Anti PD-1  Anti PDL-1  Anti CTLA-4 | No |
| Veliparib (ABT-888) | PARP 1-2 | No | Phase I/II and in animal model | Anti PDL-1  Anti CTLA-4 | No |
| BG-290 | PARP 1-2 | No | Phase I/II | Anti PD-1 | No |
