# Supplementary material for: Combining PARP Inhibition, Radiation, and Immunotherapy: A Possible Strategy to Improve the Treatment of Cancer?
Source: Int J Mol Sci. 2018 Nov 28;19(12):3793. doi: 10.3390/ijms19123793 (PMC6321381; doi:10.3390/ijms19123793)
Supplement: Supplementary file 1 [file ijms-19-03793-s001.zip › Supplementary Table 3.docx]

**Supplementary Table 3: association between external radiation therapy and immunotherapy**

Many studies are currently investigating the safety and efficiency of the association between external radiation therapy and immunotherapy. We focus in this table on the type of cancer which are also evaluating for PARP inhibitors treatment in association with either radiation therapy or immunotherapy. Stereotactic body radiation therapy (SBRT) is the main type of radiation therapy used and the immune check point inhibitors (anti PD-1, anti PDL-1, anti CTLA-4) are the most used immunotherapies (source: clinicaltrials.gov library).

| ClinicalTrials.gov Identifier | Phase | Cancer | Drugs Immunotherapy | Radiation | Other drugs |
| --- | --- | --- | --- | --- | --- |
| NCT03175666 | I/II | Triple Negative Breast Cancer | Avelumab, PDL-1 blockade | Stereotactic Body Radiation Therapy | capecitabine cisplatin cyclophosphamide 5-Fluorouracil Leucovorin nab-paclitaxel Lovaza |
| NCT03366844 | I/II | High-risk, ER-positive and HER2-negative breast cancer Triple Negative Breast Cancer | Pembrolizumab, PD-1 blockade (neoadjuvant : 2 injection) | Radiation boost 8 Gy for 3 fractions. | standard treatment |
| NCT03387085 | I/II | Triple Negative Breast Cancer (metastatic or unresecable) | Avelumab, PDL-1 blockade | Stereotactic Body Radiation Therapy | combination with others drugs |
| NCT03151447 | I | Triple Negative Breast Cancer (metastatic) | PD-1 blockade (JS001) | Stereotactic Body Radiation Therapy  (SBRT is delivered to 1~5 measurable metastatic lesions of liver, lung, bone, brain or lymph nodes in limited fractions) |  |
| NCT03277482 | I | Recurrent Gynecological Cancer Recurrent and Metastatic Cervical Cancer Recurrent and metastatic Ovarian Cancer Recurrent and Metastatic Vaginal Cancer Recurrent and Metastatic Vulvar Cancer Recurrent and Metastatic Endometrial Cancer | Tremelimumab, CTLA-4 blockade Durvalumab, PDL-1 blockade | first immunotherapy infusion or on the following day 1 day or 5 days. |  |
| NCT03312114 | II | Persistent or Recurrent Epithelial Ovarian Primary Peritoneal Fallopian Tube Cancer | Avelumab, PDL-1 blockade | Stereotactic ablative radiotherapy |  |
| NCT03262454 | II | Small Cell Lung Cancer (Recurrent) | Atezolizumab, PDL-1 blockade | hypofractionated radiation therapy  24 Gy over 4 fractions in days 1-4 of 1st cycle of atezolimumab |  |
| NCT03043599 | I/II | Small Cell Lung Cancer (extensive stage) | Nivolumab, PD-1 blockade Ipilimumab, CTLA-4 blockade | thoracic radiation therapy 30 Gy in 10 fractions |  |
| NCT02701400 | II | Small Cell Lung Cancer (Recurrent) | Tremelimumab, CTLA-4 blockade Durvalumab, PDL-1 blockade | Stereotactic Body Radiation Therapy hypofractionated radiation therapy |  |
| NCT02839265 | II | Non-small Cell Lung Cancer (advanced) | FLT3 Ligand Therapy (CDX-301) | Stereotactic Body Radiation Therapy |  |
| NCT03035890 |  | Non-small Cell Lung Cancer (metastatic) | Nivolumab, PD-1 blockade Atezolizumab, PDL-1 blockade Pembrolizumab, PD-1 blockade | Stereotactic Body Radiation Therapy 3-5 fraction course of radiation therapy, total dose 30-50 Gy |  |
| NCT03176173 | II | Non-small Cell Lung Cancer (metastatic) | Nivolumab, PD-1 blockade Atezolizumab, PDL-1 blockade Pembrolizumab, PD-1 blockade | Image Guided Radiation Therapy daily for up to 10 days (within 2 weeks) |  |
| NCT03110978 | II | Non-small Cell Lung Cancer (reccurent stage I/II) | Nivolumab, PD-1 blockade | Stereotactic Ablative Radiation Therapy |  |
| NCT03383302 | I/II | Non-small Cell Lung Cancer (stage I/II) | Nivolumab, PD-1 blockade | Stereotactic Body Radiation Therapy |  |
| NCT03313804 | II | Non-small Cell Lung Cancer | Nivolumab, PD-1 blockade Atezolizumab, PDL-1 blockade Pembrolizumab, PD-1 blockade | Stereotactic Ablative Radiation Therapy |  |
| NCT03168464 | I/II | Non-small Cell Lung Cancer (metastatic) | Nivolumab, PD-1 blockade (adjuvant) Ipilimumab, CTLA-4 blockade (with radiation) | Non-ablative Radiation Therapy 6 Gy x 5 fractions |  |
| NCT03275597 | Ib | Non-small Cell Lung Cancer (oligometastatic) | Tremelimumab, CTLA-4 blockade Durvalumab, PDL-1 blockade | Stereotactic Body Radiation Therapy |  |
| NCT00006470 | II | Non-small Cell Lung Cancer (completely resected stage II/IIIa) | monoclonal antibody 11D10 anti-idiotype vaccine monoclonal antibody 3H1 anti-idiotype vaccine |  |  |
| NCT03245177 | I | Non-small Cell Lung Cancer (locally advanced) | Pembrolizumab, PD-1 blockade | 60-66 Gy in 30-33 fractions, 2 Gy per fraction |  |
| NCT03223155 | I | Non-small Cell Lung Cancer (metastatic) | Nivolumab, PD-1 blockade Ipilimumab, CTLA-4 blockade | Stereotactic Body Radiation Therapy |  |
| NCT03224871 | I | Non-small Cell Lung Cancer (metastatic) | Nivolumab, PD-1 blockade Pembrolizumab, PD-1 blockade Intralesional IL-2 | 8 Gy x 3 fractions palliative regimen |  |
| NCT00455572 | I | Non-small Cell Lung Cancer (resected stage IB, II or IIIA, MAGE-A3-positive) | Immunotherapeutic GSK1572932A | Regimen will be based upon the site's own standard procedures | Cisplatin  Vinorelbine |
| NCT02463994 | I | Non-small Cell Lung Cancer (metastatic) | Atezolizumab, PDL-1 blockade | Hypofractionated Image-Guided Radiotherapy |  |
| NCT03169738 | I/II | Non-small Cell Lung Cancer (progression on or after treatment with PD-1/PD-L1 blockade) | Avelumab, PDL-1 blockade Nivolumab, PD-1 blockade Vaccine : ALT-803 ETBX-011 ETBX-021 ETBX-051 ETBX-061 GI-4000 GI-6207 GI-6301 haNK | Stereotactic Body Radiation Therapy | Bevacizumab Capecitabine Cisplatin cyclophosphamide 5-Fluorouracil (5-FU) fulvestrant leucovorin nab paclitaxel Lovaza Oxaliplatin |
| NCT03391869 | III | Non-small Cell Lung Cancer (metastatic) | Nivolumab, PD-1 blockade Ipilimumab, CTLA-4 blockade | Local Consolidative Therapy (LCT) : surgery or radiation therapy |  |
| NCT03237377 | II | Non-small Cell Lung Cancer (metastatic) | Tremelimumab, CTLA-4 blockade  Durvalumab, PDL-1 blockade | standard thoracic radiation (RT) (45Gy in 25 fractions) |  |
| NCT02662634 | II | Non-small Cell Lung Cancer (stage III) | AGS-003-LNG : autologous dendritic cell immunotherapy | Radiation therapy | Carboplatin Abraxane Alimta Cisplatin Taxol |
| NCT03050060 | II | Non-Small Cell Lung Cancer (metastatic) | Atezolizumab, PDL-1 blockade Nivolumab, PD-1 blockade Pembrolizumab, PD-1 blockade | Hypofractionated Radiation Therapy | Nelfinavir Mesylate |
| NCT02221739 | II | Non-Small Cell Lung Cancer (metastatic) | Ipilimumab, CTLA-4 blockade | 6 Gy x5, later changed to 9.5 Gy x3 (conformally or by intensity modulated RT (IMRT) with image guidance to maximally spare normal tissue). |  |
| NCT00879866 | I | Non-Small Cell Lung Cancer | immunocytokine EMD 521873 (Selectikine) | local irradiation (5 x 4 Gy) |  |
| NCT03446547 | II | Non-Small Cell Lung Cancer (Stage I) | Durvalumab, PDL-1 blockade | Stereotactic Body Radiation Therapy |  |
| NCT03050554 | I/II | Non-Small Cell Lung Cancer (Early stage) | Avelumab, PDL-1 blockade | Stereotactic Body Radiation Therapy :  12Gy x 4 fractions or 10Gy x 5 fractions |  |
| NCT02831933 | II | Non-Small Cell Lung Cancer | Nivolumab, PD-1 blockade | Stereotactic Body Radiation Therapy :  30 Gy : 6 Gy X 5 fractions | Valacyclovir ADV/HSV-tk |
| NCT02768558 | III | Non-small Cell Lung Cancer (locally advanced) | Nivolumab, PD-1 blockade | Thoracic Radiation therapy : High-precision radiation therapy | Cisplatin Etoposide |
| NCT02623595 | II | Non-Small Cell Lung Cancer (metastatic) | rhGM-CSF | Stereotactic Body Radiation Therapy |  |
| NCT03102242 | II | Non-small Cell Lung Cancer (Unresectable Stage IIIA and IIIB) | Atezolizumab, PDL-1 blockade | 60 Gy given in 2 Gy fractions | Carboplatin paclitaxel |
| NCT01436968 | III | Localized Prostate Cancer | ProstAtak Immunotherapy | Standard external beam radiation therapy | valacyclovir ADT |
| NCT01818986 | II | metastatic castration-resistant prostate cancer | Sipuleucel-T | Stereotactic ablative radiotherapy |  |
| NCT01903083 | I | Borderline Resectable and Locally Advanced Pancreatic Adenocarcinoma | Tadalafil (inhibiteurs de la phosphodiestérase de type 5) | 3 doses of radiation (8-10 Gy per fraction) | Gemcitabine |
| NCT01342224 | I | Borderline Resectable and Locally Advanced Pancreatic Adenocarcinoma | Tadalafil Vaccination with telomerase vaccine and GM-CSF | 3 doses of radiation (8-10 Gy per fraction) | Gemcitabine |
| NCT02648282 | II | Locally Advanced Pancreatic Adenocarcinoma | Pembrolizumab, PD-1 blockade GVAX (pancreatic cancer vaccine) | Stereotactic Body Radiation Therapy (6.6 Gy over 5 days) start during the second dose of immunotherapy (3 weeks after the first dose of immunotherapy) | cyclophosphamide |
| NCT02885727 | II | Metastatic Pancreatic Adenocarcinoma | Durvalumab, PDL-1 blockade | First lesion to receive 25 Gy / 5 daily consecutive fractions of 5 Gy Second lesion to receive15 Gy / 5 daily consecutive fractions |  |
| NCT03161379 | II | Borderline Resectable and Locally Advanced Pancreatic Adenocarcinoma | GVAX (pancreatic cancer vaccine) Nivolumab, PD-1 blockade | Stereotactic Body Radiation Therapy (6.6 Gy over 5 days) start during the second dose of immunotherapy (3 weeks after the first dose of immunotherapy) | cyclophosphamide |
| NCT01595321 | II | Resectable Pancreatic Adenocarcinoma | GVAX (pancreatic cancer vaccine) | Stereotactic Body Radiation Therapy (6.6 Gy over 5 days) | cyclophosphamide Folfirinox |
| NCT03374293 | II | Unresectable Pancreatic Cancer | PD-1 blockade | 45-50.4 Gy, 5 x per week, 1.8Gy/fx. Radiation begun the day after the first dose of anti-PD-1 antibody |  |
| NCT02311361 | II | Unresectable Pancreatic Cancer | Tremelimumab, CTLA-4 blockade Durvalumab, PDL-1 blockade | Stereotactic Body Radiation Therapy 8Gy x 1 ; 5Gy x 5 |  |
| NCT02868632 | Ib | Unresectable and non metastatic Pancreatic Cancer | Tremelimumab, CTLA-4 blockade MEDI4736, PDL-1 blockade | Stereotactic Body Radiation Therapy 6 Gy x 5 Days |  |
| NCT03127007 | I/II | Localized Rectal Cancer | Atezolizumab, PDL-1 blockade | 1.8 to 2 Gy from day 1 to 5 during 5 consecutive weeks | 5-FU based radio-chemotherapy |
| NCT02586610 | II | Localized Rectal Cancer | Pembrolizumab, PD-1 blockade | 50.4 GY in daily fractions of 1.8 Gy over a 6 week interval | Capecitabine |
| NCT03018288 | II | Gliobastoma | Pembrolizumab, PD-1 blockade | Intensity-Modulated Radiation Therapy 60 Gy : 2 Gy x 30 | Temozolomide Vaccine HSPPC-96 |
| NCT03426891 | I | Gliobastoma | Pembrolizumab, PD-1 blockade | Intensity-Modulated Radiation Therapy 60 Gy : 2 Gy x 31 | Temozolomide  Vorinostat (HDAC) |
| NCT02968940 | II | Gliobastoma (Transformed IDH Mutant Glioblastoma) | Avelumab, PDL-1 blockade | Hypofractionated radiation therapy (HFRT) 30 Gy : 6 Gy x 5 |  |
| NCT03425292 | I | Gliobastoma | Nivolumab, PD-1 blockade Ipilimumab, CTLA-4 blockade | Intensity-Modulated Radiation Therapy 60 Gy : 2 Gy x 31 (conformed radiotherapy) | Temozolomide |
| NCT00323115 | II | Gliobastoma | Dendritic Cell Vaccine | Intensity-Modulated Radiation Therapy 60 Gy : 2 Gy x 31 (conformed radiotherapy) | Temozolomide |
| NCT02336165 | II | Gliobastoma | Durvalumab, PDL-1 blockade | Intensity-Modulated Radiation Therapy 60 Gy : 2 Gy x 31 (conformed radiotherapy) | Bevacizumab |
| NCT03085719 | II | Head and Neck Cancer Squamous Cell Carcinoma (metastatic) | Pembrolizumab, PD-1 blockade | High Dose radiation will be given in 3 fractions Low Dose Radiation will be given in 2 fractions |  |
| NCT03383094 | II | Head and Neck Cancer Squamous Cell Carcinoma (locally advanced) | Pembrolizumab, PD-1 blockade | 70 Gy : 33 to 35 fractions | Cisplatin |
| NCT03313804 | II | Head and Neck Cancer Squamous Cell Carcinoma (metastatic) Non-small Cell Lung Cancer (metastatic) | Immune check point inhibitor | Stereotactic Body Radiation Therapy OR Fractionated radiation therapy |  |
| NCT03051906 | I/II | Head and Neck Cancer Squamous Cell Carcinoma (locally advanced) | Durvalumab, PDL-1 blockade | Intensity-Modulated Radiation Therapy 69,9 Gy : 2,12 Gy x 33 | Cetuximab |
| NCT03317327 | I/II | Head and Neck Cancer Squamous Cell Carcinoma (locally advanced) | Nivolumab, PD-1 blockade | Reirradiation 60 Gy (1,5 Gy fractions twice daily) |  |
| NCT02296684 | II | Head and Neck Cancer Squamous Cell Carcinoma (locally advanced) | Pembrolizumab, PD-1 blockade | Intensity-Modulated Radiation Therapy | Cisplatin |
| NCT03283605 | I/II | Head and Neck Carcinoma (metastatic) | Tremelimumab, CTLA-4 blockade Durvalumab, PDL-1 blockade | Stereotactic Body Radiation Therapy ( to 2-5 oligometastases) |  |
| NCT03169764 | I/II | Head and Neck Carcinoma (metastatic) | Pembrolizumab, PD-1 blockade Nivolumab, PD-1 blockade Vaccine : ALT-803 ETBX-011 ETBX-021 ETBX-051 ETBX-061 GI-4000 GI-6207 GI-6301 haNK | Stereotactic Body Radiation Therapy | Bevacizumab Capecitabine Cetuximab Cisplatin cyclophosphamide  5-Fluorouracil  fulvestrant leucovorin nab-paclitaxel Lovaza |
| NCT03426657 | II | Head and Neck Cancer Squamous Cell Carcinoma (locally advanced) | Tremelimumab, CTLA-4 blockade Durvalumab, PDL-1 blockade | 35 x 2.0/1.8/1.6 Gy |  |
| NCT02999087 | III | Head and Neck Cancer Squamous Cell Carcinoma (locally advanced) | Avelumab, PDL-1 blockade | Intensity-Modulated Radiation Therapy 36 x 2.0/1.8/1.6 Gy | Cisplatin Cetuximab |
| NCT02764593 | I | Head and Neck Cancer Squamous Cell Carcinoma (locally advanced) | Nivolumab, PD-1 blockade | Intensity-Modulated Radiation Therapy 36 x 2.0/1.8/1.6 Gy | Cisplatin Cetuximab |
| NCT03247712 | I/II | Head and Neck Cancer Squamous Cell Carcinoma (locally advanced) | Nivolumab, PD-1 blockade | 8Gy x 5 |  |
| NCT03267498 | II | Head and Neck Cancer Squamous Cell Carcinoma (locally advanced) | Nivolumab, PD-1 blockade | 33 x 2,12 Gy | Cisplatin |
| NCT03101475 | II | Colorectal cancer (liver metastases) | Tremelimumab, CTLA-4 blockade Durvalumab, PDL-1 blockade | Stereotactic Body Radiation Therapy : 3 X 10 Gy |  |
| NCT03169777 | I/II | Colorectal cancer (metastatic) | Avelumab, PDL-1 blockade Nivolumab, PD-1 blockade ALT-803 ETBX-011 ETBX-021 ETBX-051 ETBX-061 GI-4000 GI-6207 GI-6301 haNK | Stereotactic Body Radiation Therapy | bevacizumab capecitabine cetuximab Cyclophosphamide 5-Fluorouracil (5-FU) fulvestrant leucovorin nab paclitaxel Lovaza oxaliplatin |
| NCT03104439 | II | Colorectal cancer Pancreatic cancer | Nivolumab, PD-1 blockade Ipilimumab, CTLA-4 blockade | Radiation therapy |  |
|  |  |  |  |  |  |
|  |  |  |  |  |  |
|  |  |  |  |  |  |
