# Supplementary material for: Combining PARP Inhibition, Radiation, and Immunotherapy: A Possible Strategy to Improve the Treatment of Cancer?
Source: Int J Mol Sci. 2018 Nov 28;19(12):3793. doi: 10.3390/ijms19123793 (PMC6321381; doi:10.3390/ijms19123793)
Supplement: Supplementary file 1 [file ijms-19-03793-s001.zip › Supplementary Table 4.docx]

**Supplementary Table 4: Clinical trials evaluating the combination of PARPi and radiation in various cancers**

13 studies are currently evaluating the safety and the efficiency of the association between a PARP inhibitor and radiation therapy in ovarian cancer, triple negative breast cancer, rectal cancer, head and neck cancer, non-small lung cancer, glioblastoma, and brain metastases (source: clinicaltrials.gov library).

| ClinicalTrials.gov Identifier | Phase | Cancer | PARPi | Radiation | Others treatments |
| --- | --- | --- | --- | --- | --- |
| NCT03109080 | I | triple negative breast cancer | Olaparib | 3D conformal radiotherapy  intensity-modulated radiotherapy (IMRT) Simultaneous Integrated Boost (SIB) postoperative radiotherapy |  |
| NCT02227082 | I | Inoperable Breast Cancer | Olaparib | whole breast and regional lymph nodes will receive 23 x 2.03 Gy per fraction (total 46.69 Gy) Boost : 23 x 0.63Gy, Total dose: 61.18 Gy |  |
| NCT01477489 | I | Inflammatory or Loco-regionally Recurrent Breast Cancer | Veliparib | Standard radiation treatment (Limited to 60 Gy) |  |
| NCT01264432 | I | Peritoneal Carcinomatosis Recurrent Fallopian Tube Carcinoma Recurrent Ovarian Carcinoma Recurrent Primary Peritoneal Carcinoma | Veliparib | low-dose fractionated whole abdominal radiation therapy (LDFWAR) |  |
| NCT02921256 | II | Locally Advanced Rectal Cancer | Veliparib | Intensity-Modulated RadiationTherapy | Fluorouracil Capecitabine |
| NCT01589419 | I | Locally Advanced Rectal Cancer | Veliparib |  | Capecitabine |
| NCT02229656 | I | Head and Neck Neoplasms | Olaparib | Primary tumor and lymph nodes will receive 35 fractions of 2 Gy resulting in a total dose of 70 Gy Elective fields will receive 35 fractions of 1.55 Gy resulting in a total dose of 54.25 Gy in case a SIB technique is used, or 23 fractions of 2 Gy resulting in a total dose of 46 Gy in case a sequential boost technique is used |  |
| NCT02308072 | I | Head and Neck Neoplasms | Olaparib | Intensity-Modulated RadiationTherapy 70 Gy : 2 Gy x 35 | Cisplatin |
| NCT00649207 | I | brain metastases | Veliparib | Whole Brain Radiation Therapy |  |
| NCT01386385 | I/II | Unresectable Stage III Non-small Cell Lung Cancer | Veliparib | 3-Dimensional Conformal RadiationTherapy | Carboplatin Paclitaxel |
| NCT02412371 | I/II | Stage III Non-Small Cell Lung Cancer | Veliparib | Radiation treatment administered 30-34 days | Carboplatin Paclitaxel |
| NCT03212742 | I/IIa | Unresectable High Grade Glioma | Olaparib | Intensity-Modulated Radiation Therapy 60 Gy : 2 Gy x 30 | Temozolomide |
| NCT01514201 | I/II | Diffuse Pontine Gliomas (children) | Veliparib | Intensity-Modulated Radiation Therapy | Temozolomide |
